# Early Cancer Detection: What's Going on and What's Next

**DOI:** 10.1002/mco2.70653

**Published:** 2026-03-11

**Authors:** Emma Di Carlo

**Affiliations:** ^1^ Department of Medicine and Sciences of Aging “G. d'Annunzio” University of Chieti‐Pescara Chieti Italy; ^2^ Anatomic Pathology and Immuno‐Oncology Unit Center for Advanced Studies and Technology (CAST) “G. d'Annunzio” University of Chieti‐Pescara Chieti Italy

**Keywords:** artificial intelligence models, circulating cell‐free DNA, early cancer diagnosis, liquid biopsy, multicancer early detection tests, tumor‐associated biomarkers

## Abstract

Late‐stage cancer diagnosis and limited treatment options for advanced disease remain major contributors to cancer‐related morbidity and mortality. Blood‐based multicancer early detection (MCED) assays have consequently gained momentum as a means to shift diagnosis toward earlier, more curable stages. Despite their promise, substantial methodological, clinical, and implementation barriers hinder widespread adoption. Integrative approaches coupling multi‐omics profiling with advanced molecular imaging may improve detection accuracy and tumor localization, while risk‐adapted MCED paradigms could support more targeted, individualized screening strategies.

This article reviews the current landscape of MCED technologies, with a primary focus on circulating cell‐free DNA and circulating tumor DNA–based assays, and critically evaluates their developmental status, strengths, and limitations relative to established single‐cancer screening methods. The contribution of artificial intelligence, particularly advanced deep learning,  to improving sensitivity, specificity, and predictive performance is discussed. The potential of MCED assays to detect aggressive, currently unscreened malignancies and to address the unique challenges of pediatric cancers is examined. In addition, emerging alternative detection strategies, ongoing clinical validation efforts, regulatory requirements, and implementation considerations are reviewed. Finally, the impact of MCED testing on cancer mortality, quality of life, and healthcare systems is outlined, along with key technological trends shaping future development and clinical translation.

## Graphical abstract

Multicancer early detection (MCED) platforms have emerged as a promising strategy for the safe and effective early detection of multiple cancer types, with the potential to reduce metastatic burden and improve clinical outcomes, particularly for aggressive malignancies that lack effective population‐level screening. However, early‐stage lesions shed only minimal amounts of tumor‐derived material, resulting in weak molecular signals that constrain detection sensitivity. Advances in molecular profiling technologies and machine learning offer opportunities to enhance sensitivity, specificity, and tumor localization. This article reviews state‐of‐the‐art MCED technologies and their developmental and clinical validation status, critically evaluates their strengths and limitations, and discusses key challenges to widespread clinical adoption.

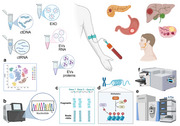

## 1 Introduction

Cancer is a leading cause of death worldwide, accounting for nearly 10 million deaths in 2020, while over 35 million new cases are expected by 2050 due to population ageing and increased exposure to risk factors related to socioeconomic development [1, 2]. Against this backdrop, early detection of cancer is critically important for lowering cancer‐related mortality and reducing the healthcare costs associated with treating advanced‐stage disease [3].

Although existing screening guidelines for breast, cervical, colorectal, lung, and prostate cancers [4] support early diagnosis, there remains an unmet need for the early detection of other tumors, such as pancreatic, bladder, kidney, liver, and hematological malignancies, which often progress aggressively and account for a large proportion of cancer‐related deaths [5, 6].

Advances in omics sciences [7], machine learning (ML) algorithms [8], and bioinformatic tools, which continue to refine sensitivity, specificity, and cancer‐type localization [9], have enabled the development of blood‐based multicancer early detection (MCED) tests. The majority of these assays analyse circulating cell‐free DNA (cfDNA) or tumor‐associated biomarkers, such as methylation or protein patterns, enabling minimally invasive early detection of multiple cancer types, including those without routine screening, well before symptoms appear [10, 11]. By enabling cancer detection at earlier, more clinically manageable stages, MCED approaches have the potential to transform oncology practice by shifting the focus from predominantly late‐stage treatment to proactive early intervention. Ongoing clinical trials are assessing the clinical performance of MCED assays, including their sensitivity and specificity, with promising results for cancers that currently lack standard screening methods.

This review synthesizes current knowledge on emerging blood‐based and biomarker‐driven approaches for early cancer detection, evaluates their clinical performance, highlights challenges and limitations, and outlines future directions. The goal is to assist researchers, clinicians, and policymakers in understanding the potential of MCED tests to enhance early cancer detection, guide personalized treatment, reduce cancer‐related mortality, and optimize healthcare resources.

Following a brief overview of MCED assay development, this review explores the contribution of AI systems in enhancing the diagnostic and predictive capabilities of MCED approaches, provides a detailed analysis of the biological factors limiting early cancer detection, summarizes relevant preclinical studies and ongoing clinical trials, and evaluates the features of minimally invasive blood‐based early detection assays relative to conventional cancer detection methods. The applicability of MCED tools in pediatric settings will also be explored, with an emphasis on the challenges and future directions for their development. Alternative early cancer detection methods, niche innovations, and emerging clinical validation efforts aimed at accelerating the real‐world implementation of MCED will be highlighted.

This review addresses the challenges associated with implementing MCED assays in healthcare systems and examines strategies to integrate them effectively into secondary prevention programs. Once fully optimized and demonstrated to provide clinical benefits beyond the current standard of care, an outcome still under investigation despite promising early evidence [12, 13, 14, 15], AI‐driven MCED approaches may advance preventive medicine, enable tumor‐specific therapies, and optimize healthcare resources through earlier, less intensive interventions.

## 2 The Short History of MCED Tests

MCED assay development began in 2014 with Guardant Health Inc.’s launch of Guardant360, a liquid biopsy that analyzes over 80 cancer‐related genes in circulating tumor DNA (ctDNA), detecting mutations, fusions, copy number alterations, and microsatellite instability (MSI) [16]. It is now primarily used in advanced cancers where tissue biopsy is impractical, particularly to guide targeted therapy in non‐small‐cell lung cancer (NSCLC) [17], breast cancer (BC) [18], colorectal cancer (CRC) [16], and other solid tumors.

In 2016, Guardant Health launched Project Lunar to extend liquid biopsy from monitoring advanced cancers to detecting early‐stage disease in high‐risk, asymptomatic individuals [19, 20]. This initiative produced the LUNAR‐1 and LUNAR‐2 assays. LUNAR‐1 targeted minimal residual disease (MRD) and post‐treatment recurrence. Guardant Health and NRG Oncology initiated the NRG‐GI005 COBRA trial to validate MRD as a biomarker for selecting stage II CRC patients for adjuvant chemotherapy. However, the trial was stopped early, as chemotherapy did not effectively clear ctDNA compared with surveillance, according to the January 2024 ASCO Gastrointestinal Cancers Symposium. LUNAR‐2 was developed for early CRC detection in average‐risk adults. Preliminary data from the pivotal ECLIPSE study, enrolling over 20,000 participants, indicate that LUNAR‐2 achieves 83% sensitivity and 90% specificity for CRC detection in adults aged 45–84 [21]. These performance results demonstrated the potential of the early CRC detection test as a reliable screening tool, which, however, should be implemented to provide a valid alternative to traditional screening methods such as faecal immunochemical test (FIT: ∼90%–95% specificity) and colonoscopies (99%–100% specificity), in early‐stage cancer diagnosis.

In 2017, Guardant Health launched the GuardantOMNI liquid biopsy test, designed for comprehensive profiling of advanced solid cancers using next‐generation sequencing (NGS) to analyze ctDNA [22, 23]. GuardantOMNI targets over 500 cancer‐associated genes and detects a broad range of genomic alterations, including single‐nucleotide variants (SNVs), such as TP53 mutations, which are common and prognostically significant; KRAS mutations affecting targeted therapy response in NSCLC and CRC; EGFR mutations prevalent in NSCLC; and BRAF mutations, which may drive resistance in EGFR‐mutant tumors. The test also identifies insertions/deletions (Indels), copy number amplifications (CNAs), and gene fusions (rearrangements), with clinically actionable examples including EGFR exon 19 deletions, BRCA1/2 alterations, HER2 amplifications, and ALK, ROS1, NTRK, and RET fusions. GuardantOMNI test also detects immunotherapy‐relevant biomarkers, including tumor mutation burden (TMB), with high TMB predicting improved responses to PD‐1/PD‐L1 inhibitors, and MSI, which identifies tumors with mismatch repair deficiency, that are often characterized by high mutational burden and responsiveness to immune checkpoint inhibitors, such as pembrolizumab [24]. These features make GuardantOMNI well‐suited to support and expedite clinical development in both immuno‐oncology and targeted therapy programs.

In 2021, GRAIL introduced the Galleri test, an MCED assay that employs ML to analyze cfDNA methylation patterns and detect more than 50 cancer types, including many that lack routine screening. Since abnormal DNA methylation occurs early in carcinogenesis and is tissue‐specific [25], it has emerged as a key biomarker for MCED testing. The Galleri test is recommended for adults at elevated cancer risk, such as those aged 50 and older, and can accurately identify the tissue of origin (TOO) of detected tumors [26], which helps guide follow‐up imaging or diagnostic tests. It is designed to complement standard cancer screening methods, such as breast, colorectal, and lung screening, enhancing early detection [27]. In the same year, the large‐scale NHS‐Galleri trial (ISRCTN91431511) began in England to assess the effectiveness of the Galleri test in detecting early‐stage cancers and reducing the incidence of stage III and IV cancer diagnoses within three to four years. The study evaluated whether an MCED test, which screens asymptomatic individuals, can reduce late‐stage cancer incidence and mortality [28]. Prospective observational studies, including SYMPLIFY (ISRCTN10226380, 2021), are providing real‐world evidence on the clinical performance of Galleri. This multicenter study assessed the sensitivity and specificity of Galleri compared with standard care in symptomatic patients referred for urgent cancer investigation [29].

After 2021, MCED tests advanced rapidly. Commercial rollout of GRAIL's Galleri test expanded with large validation studies demonstrating early‐stage detection across many cancers and prediction of TOO, informing regulatory filings for broader approval. In 2025, Exact Sciences launched Cancerguard, the first commercially available MCED test designed to detect multiple cancers by integrating multiple biomarker classes (e.g., cfDNA methylation, mutations, and fragmentomics) to improve early‐detection sensitivity. Its development is supported by DETECT‐A and ASCEND 2, the first prospective interventional MCED trials [30], with further evaluation ongoing in the FDA‐reviewed FALCON real‐world evidence registry (NCT06589310).

In 2025, Guardant Health's Shield MCD test, which is based primarily on cfDNA methylation and fragmentation patterns, received FDA Breakthrough Device designation and was selected for the National Cancer Institute's Vanguard study to evaluate its performance in large cohorts. These advances highlight growing clinical validation, regulatory progress, and early commercialization of next‐generation MCED assays. Tables 1 and 2 summarize the key features of MCED tests supported by registered prospective clinical trials with published results (ClinicalTrials.gov).

**TABLE 1:** Key characteristics of currently available MCED tests with public trial references.^a^

Test^b^	Developer/Company	Technology	Biomarkers	Cancer types covered	Focus	Approval status
CancerSEEK/Cancerguard	Johns Hopkins/Thrive Earlier Detection (USA, acquired by Exact Sciences)	cfDNA mutation panel + protein biomarkers	16 genes + 8 protein markers	Eight common cancers (ovary, liver, stomach, pancreas, oesophagus, colorectum, lung, breast)	Detects ovarian, liver, stomach, pancreatic, esophageal, colorectal, breast, and lung cancers	Not FDA‐approved or commercially available; still under clinical evaluation The DETECT‐A trial is among the key prospective studies assessing its impact
DELFI	Johns Hopkins spin‐out (USA)	cfDNA fragmentomics (fragment size, end motifs, genome‐wide patterns)	Fragmentation profiles of cfDNA	Multiple cancers	Looks for abnormal fragmentation profiles that are indicative of cancer presence	Not FDA‐approved and currently in clinical research stages
Galleri	GRAIL (Illumina spin‐off, USA)	cfDNA methylation profiling with a machine‐learning classifier	Methylation patterns across the genome (cfDNA)	>50 cancer types	Detects a signal shared by more than 50 types of cancer, including those without recommended screening tests	Not FDA‐approved or cleared; available as an LDT under CLIA, meaning only certified labs can perform it, and it must be ordered by a physician Granted FDA Breakthrough Device designation (2019), but full approval is still pending
Guardant SHIELD	Guardant Health (USA)	Targeted NGS with machine‐learning–based classifiers	cfDNA methylation patterns and fragmentation features	CRC	Blood‐based detection of colorectal cancer through combined cfDNA methylation and fragmentomic signatures	FDA‐approved for CRC screening in average‐risk adults

Abbreviations: cfDNA, cell‐free DNA; CLIA, Clinical Laboratory Improvement Amendments; CRC, colorectal cancer; DELFI, DNA evaluation of fragments for early interception; FDA, Food and Drug Administration; LDT, laboratory‐developed test; NGS, next‐generation sequencing.

^a^None of the MCED tests listed above has received broad regulatory approval as stand‐alone population screening tools. Availability and clinical use vary by country and healthcare system, with some limited to specific laboratories or research settings (cancer.org).

^b^All assays are performed using plasma samples.

**TABLE 2:** Key characteristics of currently available MCED tests with public trial references.

Test	Sensitivity	Specificity	Strength	Cost estimate	Limitations	ClinicalTrials.gov/Registry ID
CancerSEEK	Overall: ∼70% (across 8 cancer types, primarily late‐stage) Stage I Sensitivity: ∼43%	∼99%	Effective in cancers such as pancreatic and ovarian, which often lack early detection methods	No public pricing available. The test is not yet in routine clinical use	Limited evidence, mostly evaluated in research settings, with small cohorts. Benefits in terms of mortality reduction remain unproven Concern over false positives and the downstream burden of potentially unnecessary diagnostic imaging or procedures	NCT04213326 (ASCEND)
DELFI	Varies by cancer type; high sensitivity for lung cancer (∼90% for late stages)	∼99%	By employing ML and WGS, DELFI can detect subtle differences in DNA fragmentation associated with cancer, potentially identifying the presence and origin of tumors	Not available publicly. The technology is still under development	Variable sensitivity depending on cancer type, e.g., lung cancer shows high sensitivity in late stages (∼90%), but performance in other cancers or earlier stages is unclear Lack of population‐based trials showing mortality reduction or specificity in real‐world screening scenarios	NCT04825834
Galleri	Stage I: ∼16% Stage II: ∼40% Stage III: ∼77% Stage IV: ∼93% High sensitivity for pancreatic, ovarian, liver, and head/neck cancers Lower sensitivity for breast, prostate, and early‐stage colorectal cancers Correctly identifies the tissue of origin in ∼89% of positive cases	∼99.5% (very low false‐positive rate)	Has detected aggressive cancers, such as pancreatic, liver, and esophageal cancers, in early stages during real‐world use	List price: $949 per test Generally not covered by insurance; some employers or TRICARE plans may offer partial coverage Financial assistance and payment plans available, but coverage remains limited	False positives and negatives remain concerns—though specificity is high (∼99.5%), there's still potential for misdiagnosis Not meant to replace standard‐of‐care screenings (e.g., mammograms, colonoscopy); best used as a supplement Unclear clinical benefit: definitive evidence that it reduces cancer mortality or late‐stage diagnoses remains lacking	NCT05611632 (PATHFINDER) NCT05155605 (PATHFINDER 2) NCT02889978 NCT03085888 NCT03934866 NCT04241796
Guardant SHIELD	High sensitivity for colorectal cancer, including early‐stage disease, as reported in large prospective validation studies	High (reported >90%)	Organ‐specific blood‐based screening test with regulatory approval; strong clinical validation in prospective registrational studies	Not publicly available; cost may vary depending on the healthcare system and reimbursement policies	Not a pan‐cancer test; clinical utility limited to colorectal cancer screening; no evidence for mortality reduction beyond established screening strategies	NCT05117840 NCT05716477

Abbreviations: ML, machine learning; WGS, whole genome sequencing.

## 3 Role of AI in Advancing MCED Tests

In MCED testing, a key challenge is the exceptionally low signal‐to‐noise ratio of disease‐associated biomarkers in early‐stage disease. Biomarkers such as ctDNA, RNA fragments, and epigenetic alterations may occur in peripheral blood at concentrations that are close to or indistinguishable from normal physiological background variation. Detecting and interpreting these faint signals is beyond the capacity of human analysis or traditional statistical approaches alone, thereby necessitating the application of artificial intelligence (AI), particularly ML approaches.

The identification of robust cancer biomarkers generates large and highly complex datasets, making AI essential for biomarker discovery, validation, and prioritization, thereby facilitating the development of more precise MCED assays [31, 32]. AI approaches are particularly well‐suited for extracting informative patterns from high‐dimensional data. Rather than focusing on individual biomarkers, ML algorithms can simultaneously analyze thousands of molecular and clinical features, enabling the detection of multivariate biomarker signatures indicative of cancer [7]. This capability is especially critical for liquid biopsy‐based approaches, in which disease‐associated signals are sparse, heterogeneous, and obscured by substantial biological noise [33].

An additional key function of ML is the suppression of technical and biological noise and the enhancement of disease‐relevant signals. High‐throughput sequencing platforms inherently generate technical artifacts, while nonmalignant biological processes can produce molecular patterns that resemble cancer‐associated signatures. Advanced deep learning, a subset of machine learning encompassing convolutional neural networks (CNNs) and transformer‐based neural networks, is increasingly used to distinguish tumor‐derived signals from background noise, leveraging multilayered architectures that learn hierarchical representations from raw data. This discrimination improves both analytical sensitivity, by increasing true‐positive detection, and specificity, by reducing false‐positive classifications. Importantly, AI does not just flag the presence of cancer; it can also help predict the tissue or organ of origin. By learning from large reference datasets, models can map methylation patterns or fragmentomic signatures to likely anatomical sites, guiding physicians toward targeted diagnostic imaging or biopsy [34].

Beyond detection, ML supports personalized screening strategies. By integrating genomic data, lifestyle factors, and electronic health records, models can stratify individuals into different risk categories, suggesting who might benefit most from MCED testing and at what intervals [35].

Finally, AI accelerates the translation of MCED tests into clinical practice. It optimizes trial design, identifies meaningful endpoints, and enables continuous postmarket learning as more patient data accumulate.

ML approaches, encompassing supervised methods, such as random forests, support vector machines, and gradient boosting, advance MCED by efficiently identifying informative patterns in genomic and proteomic datasets [7, 36]. These models analyze labelled datasets to detect biomarkers strongly associated with cancerous signals. Deep learning is increasingly pivotal for MCED, particularly for high‐dimensional or complex biological data [37]. Architectures such as CNNs, recurrent neural networks (RNNs), and deep neural networks (DNNs) are widely applied due to their capacity to process unstructured data, including genomic sequences, medical images, and multi‐omics datasets [38]. Table 3 reports MCED data types feeding deep learning models.

**TABLE 3:** MCED data types driving deep learning models.

Data type	Example use	Deep learning model	Key references
cfDNA methylation	Methylation signatures of cancer versus healthy	CNNs, RNNs, transformers	[39, 40]
Fragmentomics	Fragment length patterns	1D CNNs	[41, 42]
Whole‐Genome Sequencing	Mutation patterns	CNNs, hybrid models	[43]
Transcriptomics (RNA‐seq)	Expression signatures	AEs, LSTMs	[44]
Proteomics/Metabolomics	Blood protein levels	DNNs, GNNs	[45]

*Note*: **AEs**: Autoencoders are used for compressing omics data. They are very powerful when the data are high‐dimensional (like methylation data, RNA‐Seq, cfDNA) and the cancers are rare compared with healthy controls (very imbalanced). **CNNs**: Convolutional neural networks learn spatial patterns in DNA or methylation sites and are used for structured genomic or epigenomic signals. 1D CNNs: One‐dimensional convolutional neural networks are a type of CNN, specifically designed to process sequential data that has one spatial, or temporal, dimension. **DNNs**: Deep neural networks can model complex cancer signals from massive data, help process noisy data, capture subtle cancer biomarkers, and are particularly useful for the tissue‐of‐origin classification component. **GNNs**: Graph neural nets can build a biological interaction graph (genes, proteins, methylation). Omics data naturally form graphs. They capture the idea that a cancer mutation or methylation change does not just happen in isolation, but impacts neighboring nodes (genes, pathways) too. **LSTMs**: Long short‐term memory networks are a type of RNN designed to handle long‐term dependencies in sequential data. **RNNs**: Recurrent neural networks have been designed for sequential data, such as DNA methylation patterns, fragmentomics, RNA expression sequences, and chromatin accessibility patterns, which have a memory component, making them well‐suited for patterns that unfold over time or positions. Transformers are used to detect mutation signatures, methylation patterns, or expression profiles predictive of early‐stage cancer, and can model global complex relationships between thousands of biomarkers to improve early cancer detection. They are currently the powerful deep learning models for a lot of structured, sequential, and high‐dimensional biological data (https://doi.org/10.1093/bioinformatics/btab083; https://doi.org/10.1038/s41746‐021‐00455‐y).

In summary, AI, particularly ML, functions as the analytical backbone of MCED, enabling the systematic interrogation of large‐scale biological datasets, the extraction of low‐abundance cancer‐associated signals, the inference of tissue or cancer origin, and the generation of clinically actionable outputs at a scale compatible with population‐level screening. Improving the efficiency of MCED requires the application of advanced statistical and analytical approaches, such as multilevel Bayesian hierarchical models (MBHMs) [46] and tissue‐specific metric frameworks (TSMFs), which enable more robust signal interpretation and TOO inference [47].

MBHM is a statistical framework that enables structured sharing of information across related groups, such as cancer types, stages, or tissue origins, while accounting for variability and uncertainty at multiple levels. By leveraging higher‐level trends across cancers or stages, the model improves parameter estimates for subgroups with sparse data, while preserving subgroup‐specific variation. Bayesian inference further provides probabilistic estimates of sensitivity, specificity, and other performance metrics, explicitly quantifying uncertainty [48]. In GRAIL's Galleri test, MBHMs have been used to share sensitivity information across tumor types and stages, particularly for rare or low‐prevalence cancers. By borrowing information from related cancers or stages, the models stabilize sensitivity estimates for subgroups with sparse data while preserving cancer‐ and stage‐specific distinctions. Although overall improvements may be limited by high intertumor heterogeneity, this approach has demonstrated utility for stage IV cancers and within‐stage analyses when low‐sensitivity cancers are excluded.

TSMFs offer a structured approach to interpret sparse, heterogeneous MCED signals by quantifying tissue‐associated molecular features, such as DNA methylation, fragmentomic patterns, or gene expression, and generating probabilistic scores that predict the TOO of circulating tumor‐derived signals. When integrated with ML classifiers, TSMF‐derived features enhance the simultaneous detection of cancer and inference of tissue origin, improving both sensitivity and specificity. The Galleri test (GRAIL) is the most extensively documented and commercially available application of TSMFs to cfDNA methylation profiles, enabling simultaneous detection of multiple cancer types and probabilistic inference of TOO to guide subsequent diagnostic evaluation [49]. This strategy also facilitates optimized trial design and provides a framework for interpreting MCED results in clinical decision‐making.

The following sections outline the biological mechanisms that impede early‐stage cancer detection and describe the advanced computational strategies developed to overcome these challenges, while critically evaluating their capabilities and limitations.

## 4 The Early Warning Signs of Tumor Onset

Tumors can release DNA into the bloodstream during apoptosis, following necrosis, or via active secretion. Resistance to programmed cell death [50, 51], a hallmark of cancer, with major molecular mechanisms summarized in Table S1, may reduce DNA release. Nonetheless, the primary factors limiting the detectability of tumor DNA are tumor burden, vascularization, and the clearance of ctDNA, as described below.

Highly aggressive tumors, that most frequently metastasize early, such as NSCLC [52], pancreatic ductal adenocarcinoma (PDAC) [53], triple negative (TN) BC [54], and melanoma [55], may be least likely to shed DNA into the circulation since both apoptotic and necrotic events are rare in their early developmental phases, enabling tumors to grow insidiously and evading immune surveillance [56].

Tumor size and growth kinetics, driven by cell proliferation and turnover rates, influence the time window during which lesions remain subclinical yet detectable [57]. Rapidly cycling cells increase apoptotic and necrotic events, enhancing the release of tumor‐derived molecules [58]. Preliminary modeling studies suggest that tumor growth dynamics influence ctDNA amounts, since a slower‐growing tumor is associated with a higher ctDNA burden than a faster‐growing tumor of the same size, which instead would require earlier intervention [59].

Vascularization, even at the precancerous stage, modulates access to the circulation [60], affecting nutrient supply and the dissemination of ctDNA, proteins, and extracellular vesicles. Early immune evasion mechanisms, such as local immunosuppression, altered antigen presentation, or clonal selection of less immunogenic cells [61], permit lesion persistence without overt inflammation, reducing symptom‐driven detection while shaping molecular signatures [62]. Finally, the clearance rate of ctDNA, which reflects the balance between its release from tumor cells and its removal from circulation, results from a complex interplay of enzymatic degradation, uptake by immune cells, intrinsic DNA properties, and organ filtration. Consequently, renal and hepatic function, DNA fragment size and structure, tumor heterogeneity, and therapeutic interventions critically modulate ctDNA levels, thereby affecting its reliability as a biomarker.

Collectively, tumor burden [63], cell death pathways [64], and ctDNA clearance dynamics regulate the kinetics of ctDNA and other tumor‐derived marker release, ultimately defining the sensitivity limits of blood‐based early detection assays.

Owing to minimal or absent neovascularization and limited capacity to breach the basement membrane or invade adjacent vasculature, most in situ lesions are unlikely to shed appreciable levels of ctDNA, thereby constraining the sensitivity of liquid biopsy–based early detection approaches. However, some in situ cancers release more DNA into the blood than others, which can result from several overlapping factors, including vascular proximity, early microvascular remodeling, high cellular turnover, a local inflammatory and enzyme‐rich microenvironment, the release of exosomes and microvesicles, defects in DNA repair pathways, and genetic mutations that render tumor cells prone to DNA fragmentation or instability, thereby increasing the likelihood of DNA shedding. These malignancies include: (1) lung adenocarcinoma in situ, which can release EGFR mutations or TP53 mutations into the blood, particularly when located near vascular structures [65]; (2) ductal in situ BC, which may release PIK3CA and TP53 mutations identifiable in ctDNA, especially in high‐grade cases [66]; (3) urothelial carcinoma in situ, which can release detectable urothelial tumor DNA in urine, and sometimes into the blood [67]; (4) premalignant colorectal lesions, such as adenomatous polyps with high‐grade dysplasia, which may release APC mutations, and serrated polyps often characterized by BRAF and KRAS mutations, and MSI [68]; (5) cervical in situ carcinoma (cervical intraepithelial neoplasia 3/high‐grade squamous intraepithelial lesion, CIN3/HSIL), which can be detected via HPV‐related ctDNA, including HPV16 or HPV18 DNA fragments [68, 69].

**FIGURE 1:**
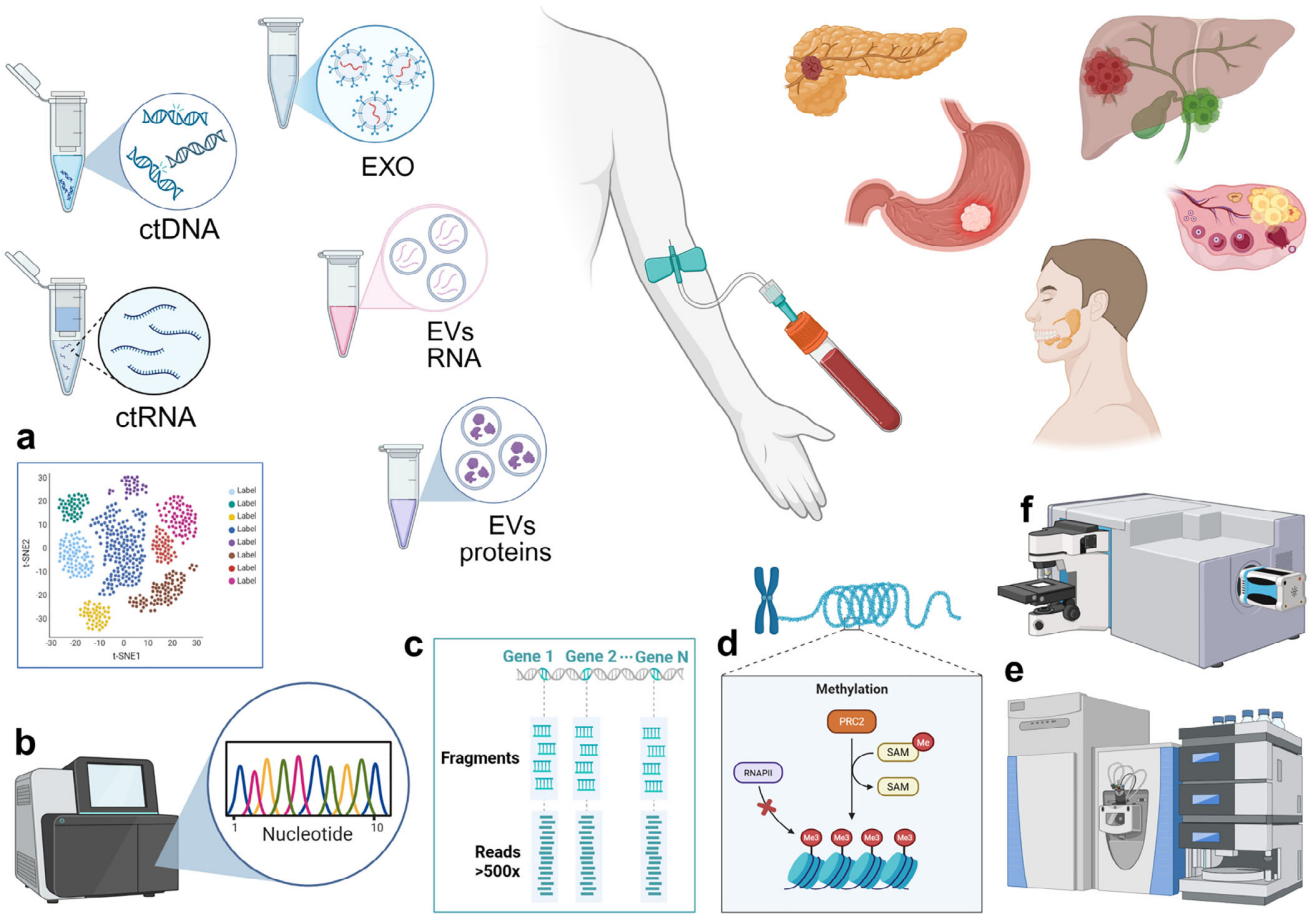
Blood‐based MCED tests are particularly valuable for identifying cancers lacking effective population screening, such as pancreatic, ovarian, esophageal, gastric, hepatocellular, cholangiocarcinoma, and head and neck cancers, as shown on the top right of the figure. Early lesions, however, shed minimal ctDNA and other tumor‐derived components, including ctRNA, exosomes, and extracellular vesicles carrying RNA or proteins, producing weak molecular signals that challenge early detection. Emerging technologies combined with ML models can enhance sensitivity, specificity, and tumor localization. The best‐performing approaches include (a) single‐cell RNA‐seq, to detect early transcriptomic alterations in cells from preneoplastic lesions; (b) NGS of ctDNA, enabling sensitive mutation detection and monitoring of tumor‐derived genetic material; (c) cfDNA fragmentomics, which analyzes characteristic fragmentation patterns; (d) DNA methylation profiling, leveraging epigenetic alterations that are more consistent than somatic mutations in early neoplasia; (e) mass spectrometry, to analyse exosomal RNA and protein profiling; (f) Raman spectroscopy, which captures early biochemical changes in biofluids involving nucleic acids, protein secondary structure, lipid composition, and metabolites. *Source*: Created with BioRender.com. (Licence number: YTXLSTIU‐0003).

Notably, ctDNA release is not determined solely by whether a tumor is in situ or invasive, but rather by its degree of biological “leakiness,” encompassing histological, genetic, and immunological characteristics. The combination of these features enables some in situ lesions to release DNA into the bloodstream early, before progression to invasive disease. A diagnostic gap persists for early neoplastic lesions that produce weak molecular signals at onset (Figure 1). To address this limitation, a range of complementary methodological approaches may be employed.

First, risk‐enriched population selection, based on germline genetic predisposition [70], environmental exposures [71, 72], or premalignant conditions, increases the pretest probability and improves the positive predictive value of early cancer detection assays. Genetic risk tests are particularly valuable for selective enrolment in the MCED tests for subjects at high risk of cancers that lack effective population‐wide screening strategies. For pancreatic cancer, germline testing for pathogenic variants in BRCA2, CDKN2A, PALB2, STK11, and ATM identifies high‐risk individuals who benefit from structured surveillance [73, 74]. In ovarian cancer, carriers of BRCA1/BRCA2 or RAD51C/RAD51D variants are enrolled in enhanced monitoring or risk‐reducing strategies due to the lack of reliable early detection tools [75, 76]. For oesophageal and gastric cancers, testing for CDH1 mutations enables identification of individuals at high risk for hereditary diffuse gastric cancer [77] while variants in TP53 and DNA repair genes can inform monitoring for Barrett's‐associated oesophageal adenocarcinoma [78]. In hepatocellular carcinoma, genetic risk stratification based on HFE variants, associated with hereditary hemochromatosis [79], SERPINA1 variants, associated with alpha‐1 antitrypsin deficiency, supports the implementation of targeted biomarker surveillance strategies [80]. For cholangiocarcinoma, germline predisposition involving genes, such as BAP1 and DNA damage repair genes, identifies subgroups warranting close hepatobiliary monitoring [81]. Finally, in head and neck cancers, germline variants in DNA repair (e.g., Fanconi anemia genes) [82] or carcinogen metabolism pathways [83, 84] identify individuals at elevated risk [85].

Second, multianalyte and multi‐omic approaches that integrate ctDNA, DNA methylation patterns, fragmentomics, circulating RNA, proteins, metabolites, extracellular vesicles, and immune‐derived signals enhance sensitivity by capturing diverse biological consequences of early tumorigenesis beyond low‐abundance mutations [85, 86]

Third, longitudinal sampling with intraindividual baselining, using repeated measurements from the same individual and their own prior values as reference, enables detection of subtle temporal deviations from an individual's molecular norm, improving early discrimination of malignant changes from baseline biological variability [87, 88].

Fourth, ultrasensitive analytical technologies, including error‐corrected sequencing [89], single‐molecule detection [90], and signal amplification strategies [91], reduce technical noise and permit reliable detection of extremely low‐frequency tumor‐derived signals.

Fifth, tumor‐adjacent and tissue‐informed biomarkers, such as field cancerization–associated epigenetic alterations or immune response signatures, provide indirect yet amplified indicators of early lesion presence [92, 93].

Finally, advanced computational modeling, incorporating ML [94], Bayesian inference [95], and prior biological knowledge, can integrate weak, heterogeneous signals across analytes and time points to infer early neoplastic states.

These approaches collectively address biological and technical limitations that underlie poor molecular signal shedding in early cancer, enabling targeted monitoring of high‐risk individuals and facilitating early, potentially curative interventions with reduced advanced‐stage disease.

## 5 Preclinical Studies and Ongoing Clinical Trials

Before clinical translation, rigorous preclinical studies are essential to ensure accuracy, reproducibility, and safety. These studies provide the foundation for MCED development by characterizing biomarker performance across diverse cancer types and stages; assessing specificity against noncancer conditions to minimize false positives; optimizing assay sensitivity for early‐stage disease detection; and establishing analytical validity through controlled laboratory experiments and retrospective cohort evaluations. Carefully designed preclinical investigations are essential to refine test performance, reduce translational risks, and generate the evidence base required for clinical trials. Table S2 summarizes studies on assay discovery (targeting ctDNA mutations, cfDNA methylation, proteins, miRNAs, or combinations), analytic development, and early validation in archived human plasma, cell lines, or animal models, representing the preclinical phase before large prospective screening trials. Many high‐impact MCED programs, such as CancerSEEK and GRAIL/CCGA, progressed from these preclinical case‐control/discovery datasets into larger prospective validation and screening trials [96]. Since case‐control/archived‐sample results can overestimate performance relative to prospective screening, this field of research emphasizes the stepwise progression from discovery to analytic validation, to prospective clinical evaluation. Kisiel's [97] commentary in *Cancer* outlines the recommended framework for developing and validating MCED tests.

By expanding beyond organ‐specific screening paradigms, MCED technologies aim to identify cancers earlier, when treatment is most effective, and to fill major gaps for cancers that currently lack population‐based screening. However, translating this promise into clinical practice requires rigorous evaluation through clinical trials that can establish not only analytic validity, but also clinical utility and real‐world feasibility [97]. The landscape of MCED trials is rapidly evolving, encompassing a spectrum of study designs from large randomized controlled trials (RCTs) to pragmatic registry‐based evaluations that embed research within existing healthcare systems and workflows. High‐profile examples include the National Health Service (NHS)‐Galleri trial, in the UK, the largest population‐based RCT to date [13, 98]; the Falcon Registry led by Exact Sciences to generate real‐world evidence (RWE) in the US healthcare systems (NCT06589310); and the National Cancer Institute (NCI)'s Vanguard Study, in the USA, a pilot under the Cancer Screening Research Network (CSRN) testing the feasibility of multiple MCED platforms in preparation for definitive mortality‐endpoint trials [99]. Alongside other industry‐sponsored and investigator‐initiated studies, these efforts are generating the evidence needed to assess whether MCED can reduce late‐stage cancer incidence and, ultimately, lower cancer‐specific and overall mortality. Table 4 illustrates the current landscape of MCED testing in ongoing clinical trials. What clearly emerges from the data in Table 4 is that: (a) “large, registrational MCED studies are concentrated among a small number of vendors,” namely GRAIL (Galleri), Guardant (Shield), ClearNote Health (Avantect), and Exact Sciences (registry‐based programs), alongside several academic or NIHR‐funded trials evaluating alternative signal modalities (e.g., Proteotype's Enlighten study); (b) “the NCI's Vanguard Study (CSRN) represents an important coordination effort,” piloting the design and execution of randomized effectiveness trials for MCED tests and, for this purpose, has selected two assays, Avantect and Shield. This pilot is expected to inform the design of future mortality‐endpoint randomized controlled trials; and (c) to balance analytical and clinical validation with demonstration of real‐world benefit, many programs adopt a two‐tier evidence strategy, in which observational studies or registries and interventional registrational studies establish test performance and safety, followed by larger pragmatic studies or RCT pilots to evaluate clinical utility, implementation, and impact on mortality [96].

**TABLE 4:** State‐of‐the‐art MCED testing in active clinical trials.

Trial name	Test/Sponsor	Registry ID(s)	Design and phase	Target enrollment/population	Primary outcome(s)/Goal(s)
REFLECTION	Galleri (GRAIL)	NCT05205967	Prospective, noninterventional cohort (RWE)	∼17,000 (individuals who opted for Galleri testing in routine care)	Understand real‐world performance, care pathways, utilization, patient/provider experiences
REACH	Galleri (GRAIL)	NCT06603259	Real‐world/Medicare‐focused prospective study	Medicare‐eligible population (older adults)—size variable per substudies	Evaluate clinical impact, safety, and implementation among Medicare‐eligible patients
Falcon	Exact Sciences MCED test	NCT06589310	Prospective real‐world evidence registry	Up to ∼25,000 (exact Sciences target) versus comparator cohorts; ages ∼50–80	Track adoption, downstream diagnostic workup, outcomes, and health services impact of Exact Sciences' MCED in routine care
Vanguard	Avantect (ClearNote) and Shield (Guardant) selected	NCT06995898	Feasibility pilot (Cancer Screening Research Network) — multiassay; cohort ∼24,000	Up to ∼24,000 adults 45–75; asymptomatic	Feasibility to run large RCTs — assess logistics, acceptability, prelim performance of Avantect and Guardant Shield in a harmonized pilot to design definitive trials
MODERNISED	Enlighten (Proteotype Diagnostics)	ISRCTN17299125	Prospective diagnostic validation (training + validation)	Early target recruitment (hundreds—trial reached >450 milestone July 2025)	Train/validate protein/immune‐response signature to detect 10 tumor types; produce data to design larger RCT

*Note*: **NCT/ClinicalTrials.gov**: National Clinical Trial (NCT) registry. It is US‐based and managed by the US National Library of Medicine (NLM) at the NIH. It assigns a unique NCT number to clinical trials, tracks studies worldwide, promotes transparency, and supports regulatory compliance. **ISRCTN Registry**: International Standard Randomised Controlled Trial Number registry. It is UK‐based and managed by BioMed Central. It assigns a unique identifier to clinical trials of any design and ensures transparency and public trial‐tracking. RCT, randomized controlled trial.

Key examples of these programs are reported in Table S3. This tiered approach reflects both the novelty and complexity of MCED. Early‐phase registries ensure the robustness and safety of testing, while larger pragmatic or randomized trials are essential to determine whether MCED adoption delivers meaningful population health benefits.

Despite the enthusiasm, key uncertainties remain, which regard how to measure meaningful benefit, how to minimize harm, such as false positives and overdiagnosis, and how to integrate MCED into existing screening pathways [100]. The current wave of trials is thus pivotal, not only for evaluating specific test performance, but also for shaping the scientific, clinical, and policy framework for this new era of cancer screening.

By mid‐2025, MCED development had largely consolidated around cfDNA methylation profiling as the predominant analytical signal, while fragmentomics, ultrasensitive sequencing technologies, and multimodal strategies integrating DNA with proteins or other analytes showed rapid methodological advancement [101, 102]. Several MCED assays have reported pivotal or registrational data, most notably updates from Galleri (PATHFINDER‐2) and Guardant Shield, indicating increasing analytical maturity and readiness for large‐scale evaluation. Concurrently, large pragmatic and registry‐based trials, including NCI‐backed initiatives, are enrolling to assess population‐level implementation and effects on stage shift, addressing the need to move beyond test accuracy toward evidence of clinical benefit. These efforts are enabled by advances in assay enrichment and error suppression, AI‐based TOO assignment and risk calibration, and study designs aligned with real‐world diagnostic workflows. Building on this progress, ongoing challenges and opportunities include the integration of emerging technologies and platforms, as well as alternative early cancer detection approaches and niche innovations that may complement or extend current MCED capabilities.

### 5.1 Emerging MCED Technologies and Platforms

#### 5.1.1 EPISEEK

The “Precision Epigenomics” Liquid Biopsy‐based MCED test, which analyzes hypermethylated DNA loci, common epigenetic biomarkers across more than 60 types of cancer. Validation data for the EPISEEK test have been shared at the 2025 American Society for Clinical Oncology (ASCO) annual meeting, where it has been reported an early‐stage sensitivity of Stage I/II ∼45%, Stage IV ∼74%; specificity: 99.5%; positive predictive value (PPV): 64.9%; negative predictive value (NPV): 99.5%; detection threshold: < 0.1 ng cfDNA for most biomarkers. Results delivered in ∼2–3 days [85, 103].

#### 5.1.2 Exact Sciences' MCED (Cancerguard EX)

A modeling study presented at ASCO 2025 suggested that annual MCED testing might reduce late‐stage diagnoses by over 40% and mortality by up to 18%, particularly in high‐risk groups. The upcoming Falcon Registry will prospectively enrol 25,000 individuals, compared with a 50,000‐person standard‐care cohort, to evaluate real‐world effectiveness and user experience [104].

#### 5.1.3 Harbinger Health's Reflex MCED

Presented at ASCO 2025, Harbinger's ctDNA‐methylation‐based MCED employs a two‐tier reflex testing paradigm: an initial methylome profiling assay optimized for high sensitivity to effectively rule out disease, followed by a confirmatory reflex test using an expanded methylation panel to improve PPV, confirm cancer presence, and identify the TOO. This strategy demonstrated meaningful early‐stage sensitivity and per‐cancer PPV, particularly in high‐risk populations, such as individuals at increased risk for obesity‐associated cancers [105].

#### 5.1.4 Guardant Health's Shield MCD (Multicancer Detection)

At the 2025 American Association for Cancer Research (AACR) Annual Meeting, Guardant Health spotlighted its Shield blood‐based, ctDNA methylation‐driven MCD assay, capturing epigenomic alterations that are common across many tumor types and often precede detectable genetic mutations during tumorigenesis. Its selection for the NCI's Vanguard Study underscores advances in liquid biopsy platforms for multicancer detection [106].

#### 5.1.5 New Cost‐Efficient, Two‐Step MCED Strategy by SeekIn

SeekIn developed a two‐step cancer screening approach: (1) OncoSeek, an affordable, blood‐based assay for broad cancer detection that uses a panel of seven protein tumor markers (PTMs: AFP, CA125, CA15‑3, CA19‑9, CA72‑4, CEA, CYFRA21‑1), together with AI‑driven pattern recognition to detect multiple cancers and estimate the probability of cancer (POC). If a cancer signal is detectd, it also outputs a TOO prediction; (2) SeekInCare, a confirmatory pan‐cancer test for individuals with positive OncoSeek results, integrating genomic, epigenetic, and proteomic features to improve specificity and refine cancer risk assessment. This strategy is designed to enhance specificity, maintain high sensitivity, and optimize cost‐effectiveness for population‐level screening while supporting clinical validation [107, 108].

### 5.2 Alternative Early Cancer Detection Methods and Niche Innovations

#### 5.2.1 Metabolic Biomarker‐Based Detection

This is a metabolic MCED method, developed at Chalmers University of Technology (Sweden), that uses free glycosaminoglycans (GAGs) (long sugar chains involved in cell communication and extracellular matrix structure) as biomarkers. Cancer reprograms cellular metabolism and extracellular matrix turnover, leading to measurable alterations in circulating GAG profiles (GAGome) in the bloodstream. The method detects 14 cancer types and identifies approximately twice as many Stage I tumors as DNA‐based tests, while requiring lower sample volumes and cost [109]. By capturing cancer‐associated metabolic alterations independent of genetic mutations, this approach enables earlier detection, including in cancers that shed little circulating DNA, such as brain and genitourinary tumors [110].

#### 5.2.2 Protein Signature Test, Enlighten

This method is currently under evaluation in the UK (the MODERNISED trial—NIHR207538). Unlike most multicancer blood tests that rely on ctDNA, Enlighten detects protein‐level signatures, measured via colored light emission, that reflect the immune system's early response to cancer, even when tumors are too small to release detectable DNA into the bloodstream. This approach has the potential to improve early‐stage detection accuracy. The test targets 10 common solid tumor types: bladder, breast, colorectal, lung, melanoma, oesophageal, ovarian, pancreatic, prostate, and renal cancers [110, 111, 112].

Noteworthy, promising single‐cancer “early detection” innovations include methods, such as (1) “PAC‐MANN” developed at Oregon Health and Science University in Portland (USA) for early pancreatic cancer detection, which uses magnetic nanosensors and a fluorescent readout to capture protease activity linked to pancreatic cancer with a sensitivity of ∼62% (early‐stage), rising to ∼85% when combined with CA 19‐9 marker, and high specificity (96–98%) [113]; (2) “IIT‐BHU's Portable Osteosarcoma Sensor”, a miniaturized, reagent‐free, self‐reporting diagnostic device developed at Indian Institute of Technology, Uttar Pradesh (India) to detect early‐stage osteosarcoma with high accuracy, by targeting a pivotal biomarker, osteopontin, associated with this malignant bone tumor [114]. The sensor's portability, rapid readout, and low cost make it suitable for resource‐limited or rural settings, with smartphone integration in development to facilitate data capture and remote monitoring.

Although recent reviews highlight the potential of MCED to transform cancer screening by enabling the detection of currently unscreened cancers, improving efficiency, lowering treatment costs, and enhancing quality of life, substantial implementation and cost barriers remain [115]. Key challenges include quality control, regulatory approval, reimbursement, and stakeholder education to ensure equitable access [116]. Radiologists remain pivotal in interpreting MCED results, confirming findings, and shaping diagnostic protocols for imaging follow‐up and policy support [117]. Ongoing large‐scale trials in the USA and UK are expected to provide critical real‐world outcome data [118, 119].

## 6 A Minimally Invasive Blood Test Versus Traditional Cancer Detection Tools

A limited subset of cancers detected by MCED tests may be broadly aligned with established screening frameworks, such as the Wilson & Jungner criteria and the Updated WHO Screening Principles (2008), but only under specific conditions. Cancers with a well‐defined “screening window” (a preclinical or early stage during which timely detection and intervention can improve survival or clinical outcomes) and established, evidence‐based screening programs, such as colorectal, breast, cervical, and lung cancer in high‐risk populations, are most likely to meet these criteria when identified by MCED testing [120, 121]. However, MCED‐based detection has not yet been demonstrated to be equivalent or superior to established single‐cancer screening modalities, including colonoscopy, mammography, HPV testing, and low‐dose computed tomography (CT). Therefore, adherence is determined by the cancer type rather than the MCED technology itself. By contrast, many rare, aggressive, or poorly characterized cancers detected by MCED tests, such as pancreatic, ovarian, biliary cancers, and certain sarcomas, currently do not meet key screening principles, particularly regarding demonstrated clinical benefit from early detection, clearly defined management pathways, and a favorable balance of benefits versus harms [122].

**FIGURE 2:**
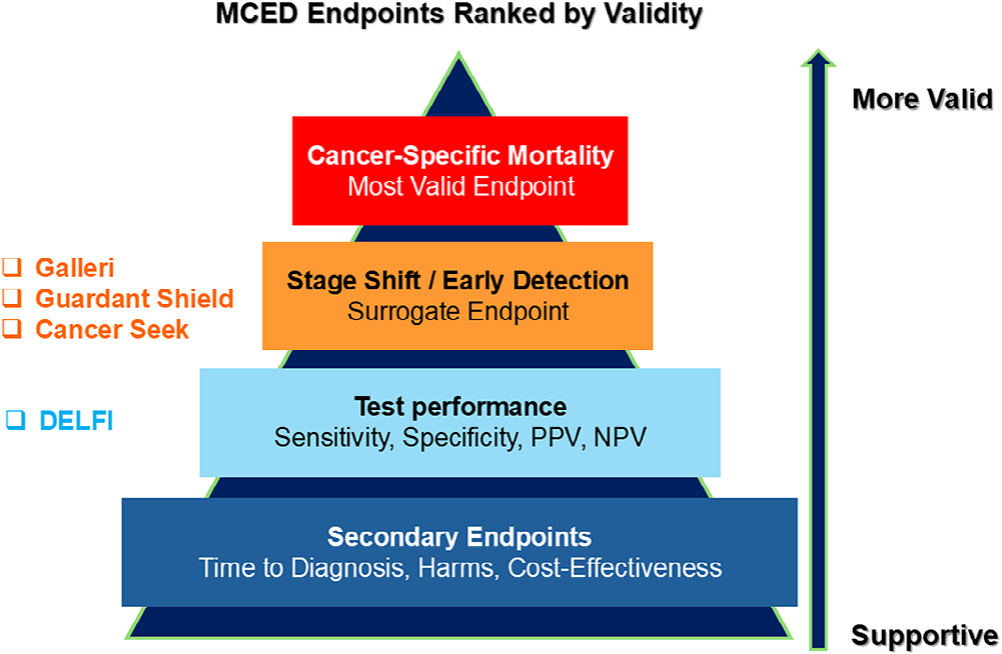
MCED endpoints ranked by validity orders trial outcomes by the strength and reliability of evidence, prioritizing reproducible, clinically meaningful, and statistically robust endpoints over exploratory or less‐validated measures. In this context, tests such as DELFI, Galleri, Guardant Shield, and CancerSEEK are evaluated against these criteria, enabling direct comparison of their performance, reliability, and evidentiary support. Higher‐ranked endpoints reflect stronger validation, guiding interpretation, and prioritization of MCED test results, in both research and clinical practice.

For the regulatory assessment and scientific validation of MCED assays as population screening tools, the most definitive endpoint is a reduction in cancer‐specific mortality. Surrogate endpoints, such as stage shift, may provide supportive evidence, but cannot substitute for direct demonstration of clinical benefit. As of early 2026, no MCED assay has yet demonstrated a statistically significant reduction in cancer‐specific mortality in randomized controlled trials. While technical performance metrics, such as sensitivity and specificity, are essential for validation, they cannot demonstrate clinical utility without evidence from trials specifically designed to evaluate effects on patient outcomes, including stage shift and survival. Figure 2 displays the MCED assays listed in Tables 1 and 2, ranked by the clinical endpoints achieved in their respective studies registered on ClinicalTrials.gov.

The sensitivity and specificity of MCED tests are crucial to how effective and trustworthy these tests are in clinical practice, and depend on the test type, the cancer stage, and the tumor's biological characteristics. Most current MCED tests prioritize high specificity to minimize false positives, which can lead to unnecessary anxiety and medical procedures, while achieving reasonable sensitivity for detecting multiple cancers at different stages [115]. Early‐stage cancers, especially stage I, are more challenging to detect, with sensitivity often below 50%, as highlighted in Tables 1 and 2. Among current MCED tests, Galleri has the most robust cost‑effectiveness modeling to date and can be cost‑effective at current prices in some healthcare systems. CancerSEEK‑derived tests show promise given lower price points, but economic evidence is still emerging. PanSeer and DELFI currently lack comprehensive published cost‑effectiveness models, so their value will become clearer when more real‑world implementation data are available.

For common cancers such as breast, cervical, colorectal, and prostate, which already have established, effective screening programs that detect many cases early, MCED tests may offer limited additional benefit. In contrast, for aggressive, often asymptomatic cancers, such as pancreatic, liver, ovarian, kidney, and brain cancers, that currently lack reliable screening methods and are typically diagnosed at advanced stages, MCED tests are expected to be more effective by providing early diagnosis with a meaningful impact on patient survival. However, key technical and clinical challenges must be overcome to achieve this goal, such as: (a) Low tumor fraction in early disease, resulting in low ctDNA concentrations that limit sensitivity, particularly for brain, pancreatic, and certain liver and kidney lesions. This represents the primary biological constraint [86]. (b) Heterogeneous biology across tumor types. Tumor shedding rates, methylation signatures, and protein expression vary widely between and within cancer types, meaning a single assay may underperform for certain cancers [115]. (c) Demonstration of clinical utility by trials showing that earlier detection changes management and improves outcomes, reducing mortality or improving quality of life, at an acceptable cost. Retrospective case‐control performance often overestimates real‐world performance.

Actionable solutions to overcome these challenges involve, first and foremost, technical and biomarker improvements, including: (a) Development of multimodal assays, which combine methylation, fragmentomics, targeted mutation panels, and protein biomarkers. Multimodal data increases sensitivity for low‐shedding tumors, while retaining specificity [86]. (b) Use of ultra‐deep sequencing with error suppression, incorporating molecular barcodes and duplex sequencing, which enhances molecular depth and enables reliable detection of very low variant allele fractions. This approach is particularly effective for cancers with low ctDNA levels, such as pancreatic and brain tumors [86]. (c) Development of TOO modeling, which invests in richer training sets including rare and underrepresented tumor subtypes to improve ML models that predict where a positive signal originates, to reduce downstream diagnostic burden. Real‐world data (e.g., PATHFINDER and other registry studies) are important for refining TOO models. (d) Integration of clinical priors, combining test results with patient risk factors, such as age, lifestyle (smoking, diet, obesity), exposure to radiation or chemicals, workplace hazards, viral (Human Papillomavirus, Hepatitis B and C, Eptein‐Barr virus), bacterial (Helicobacter pylori), or parasitic (Schistosoma haematobium) infections, family history, and germline risk variants, to generate context‐aware posttest probabilities and recommended diagnostic pathways. This approach minimizes unnecessary imaging in individuals with low pretest probability. Table S4 outlines cancer‐specific challenges and tailored solutions to improve early detection of hard‐to‐detect tumors.

In the short term, early detection of aggressive tumors could benefit from integrating MCED tests, which have limited tumor‐localization precision, with conventional detection methods. Imaging techniques, including magnetic resonance imaging (MRI), CT, positron emission tomography (PET), and ultrasound, combined with biopsy and subsequent immuno‐molecular characterization, remain the gold standard for confirming cancer, identifying molecular markers, and guiding therapy [88]. A combination of both approaches implemented with advanced molecular imaging methods, primarily molecular MRI, PET, or single‐photon emission computed tomography (SPECT)‐based techniques, may provide the most comprehensive cancer detection and management strategy.

As of now, no MCED tests have been approved by the US FDA or the European Medicines Agency (EMA). Nevertheless, several MCED assays are available in the USA as laboratory‐developed tests (LDTs) regulated under the Clinical Laboratory Improvement Amendments (CLIA). In July 2024, the FDA approved Guardant Health's blood‐based shield test for CRC screening in average‐risk adults aged 45 years and older, representing an important milestone in noninvasive cancer detection [21]. In parallel, to expedite the development and regulatory review, the FDA has granted breakthrough device designation to selected MCED tests, including the OverC multicancer detection blood test from Burning Rock Biotech, which analyzes cfDNA methylation patterns using NSG and ML [122, 123], to identify multiple cancer types (supported by THUNDER case‐control study).

## 7 Current Studies and Prospects for Early Detection of Childhood Tumors

In adults, several MCED tests have been developed and are currently undergoing clinical evaluation, including the Galleri test by GRAIL [124], the Guardant Shield test by Guardant Health, and emerging platforms, such as PANAROMIC by Dxcover. This is a next‐generation liquid biopsy technology, with substantial clinical evedence, that integrates attenuated total reflectance infrared (ATR‐IR) spectroscopy with AI algorithms to generate a comprehensive multi‐omic spectral profile of blood, enabling early detection of brain, colorectal, lung, and pancreatic cancers [125]. Compared with their level of development for adults, the application of MCED tests in pediatric oncology is currently limited. Pediatric cancers, (the most frequent include leukemias ∼30%, brain and central nervous system tumors ∼26%, lymphomas ∼10%, neuroblastoma ∼6%, Wilms' tumor ∼5%, rhabdomyosarcoma ∼3%, retinoblastoma ∼2%, osteosarcoma and Ewing sarcoma ∼3–4%) [126] often differ biologically from adult cancers, presenting unique challenges for early detection. Several factors make the development of MCED tests in children a complex task. Psychological and ethical considerations are central to implementing pediatric MCED tests. While early detection offers potential clinical benefits, testing children may provoke anxiety, uncertainty, and altered self‐perception in patients and families, particularly when results are ambiguous or predictive rather than diagnostic. Ethical challenges include informed consent, assent, and risk of overdiagnosis. Ensuring age‐appropriate communication, involving guardians in decision‐making, and providing robust psychosocial support are essential for mitigating distress and upholding ethical standards. This underscores that the deployment of pediatric MCED must integrate clinical, ethical, and psychosocial frameworks. Table 5 lists the key challenges and potential solutions to implement MCED tests for pediatric use.

**TABLE 5:** Challenges and solutions in the development of pediatric MCED tests.

Challenge	Details	Potential solutions	Key references
Rarity and heterogeneity of pediatric cancers	Pediatric cancers are rare, biologically diverse, and distinct from adult tumors (embryonal origin, developmental pathways). These features complicate biomarker discovery	Target high‐risk groups (genetic predisposition, family history). Establish large multicenter biobanks	[127]
Different mutational landscapes	Pediatric tumors generally harbor fewer somatic mutations and less mutational burden than adult cancers, making mutation‐based detection harder	Use epigenetic, transcriptomic, and proteomic biomarkers (e.g., cfDNA methylation, fragmentomics) instead of mutation‐only approaches	[127]
Low ctDNA levels	ctDNA levels may be very low in children due to smaller tumor burden or slower cell turnover, challenging sensitivity	Develop ultra‐sensitive assays (UMI‐based NGS, digital PCR). Explore nonblood biofluids (urine, CSF, saliva)	[128]
Age‐related physiological variation	Developmental processes (organ growth, hematopoiesis, hormones) can generate background signals that complicate distinguishing cancer‐derived signals	Build age‐specific reference datasets to filter normal developmental signals	[129, 130]
Assay sensitivity and specificity	The need to detect very low tumor signals while minimizing false positives	Combine multi‐omics signals; apply machine learning for noise reduction; validate in prospective cohorts	[131]
Sample collection limitations	Minimally invasive sampling is critical in pediatrics, but blood volume is limited. Optimizing tests to work with low‐input material is essential	Optimize assays for low‐input material; develop finger‐prick or dried blood spot tests	[132]
Multi‐omics integration	Pediatric cancers may require combined approaches (cfDNA methylation, fragmentomics, transcriptomics, proteomics) to achieve reliable detection	Integrate cfDNA, methylation, transcriptomics, and proteomics for higher accuracy	[133]
Defining target populations	Pediatric cancer incidence is low; population‐wide screening may not be feasible. Risk stratification (genetic predisposition syndromes, family history) may be needed	Implement targeted screening in high‐risk groups (e.g., Li‐Fraumeni, familial syndromes)	[134]
Uncertain clinical utility	Evidence is lacking on whether early detection in asymptomatic children improves outcomes, since many pediatric cancers are aggressive and progress rapidly	Conduct longitudinal studies to assess impact of early diagnosis on survival and morbidity	[135]
Incidental findings/overdiagnosis	Risk of overdiagnosis or detection of indolent tumors that may never cause harm, leading to overtreatment	Develop clinical guidelines for interpreting and managing incidental findings	[135]
Psychological and ethical considerations	False positives cause distress for families and raise ethical concerns around pediatric testing	Provide genetic counseling, psychological support, and clear communication pathways	[136]
Consent and assent issues	Parents provide consent, but children's assent and data ownership raise ethical questions	Establish pediatric‐specific consent frameworks and long‐term governance of genomic data	[137]
Regulatory and trial design hurdles	Trials are difficult due to small populations and ethical constraints	Use adaptive trial designs, global collaborations, and registry‐based trials	[138]
Cost‐effectiveness	Rare cancers make broad screening potentially cost‐inefficient	Focus on targeted subpopulations; perform health‐economic modeling	[139]
Equity and access	Risk of disparities in access across regions and populations	Ensure diverse cohort inclusion; develop affordable and scalable technologies	[140]

Abbreviations: CSF, cerebrospinal fluid; NGS, next‐generation sequencing; UMI, unique molecular identifiers.

Nevertheless, certain technologies show promise in this area. Epitope detection in monocytes (EDIM) technology exploits the innate phagocytic activity of activated CD14^+^/CD16^+^ monocytes to detect tumor‐specific antigens circulating in the bloodstream. These immune cells internalize tumor‐derived epitopes, including Transketolase‐like 1 (TKTL1), associated with altered glucose metabolism and the Warburg effect; Apo10 (DNaseX epitope), linked to apoptosis resistance and DNA fragmentation; and disialoganglioside (GD2), a tumor‐specific marker for neuroblastoma. Flow cytometry can then be used to measure tumor epitope expression levels and heterogeneity across monocyte subsets and, when combined with multiparameter staining, to visualize their activation states. This noninvasive liquid biopsy method has been applied for the early detection, monitoring of treatment response, and detection of recurrence in pediatric tumors, notably rhabdomyosarcoma [141] and neuroblastoma [142], by identifying tumor epitopes in activated monocytes [143], thus avoiding radiation exposure, which is important in pediatrics. It offers a complementary method, alongside imaging and molecular diagnostics, that is especially valuable in screening high‐risk children (e.g., with genetic predispositions or familial cancer syndromes). Most studies report an EDIM score = (% CD14^+^/CD16^+^ positive monocytes) × 10, with ROC‐derived cut‐off values to define a “positive” result [141, 144]. Tumor biomarkers that have been validated in pediatrics and are detectable by EDIM, with available quantitative performance metrics, are described in Table 6. Further large‐scale studies are necessary to validate EDIM's clinical utility and to standardize its application across various pediatric tumor types.

**TABLE 6:** EDIM performance for early detection of pediatric tumor biomarkers.

Biomarker	Biological function and diagnostic rationale	Evidence in neuroblastoma	Evidence in rhabdomyosarcoma	Notes
TKTL1	TKTL1 is a key enzyme linked to nonoxidative PPP flux, Warburg metabolism, invasion, and therapy resistance; frequently overexpressed in solid tumors [145, 146]. In EDIM, TKTL1 positivity reflects uptake of tumor material with glycolytic/metabolic reprogramming signatures [147] TKTL1 has proven to be more sensitive and specific than traditional tumor markers for early cancer detection [148]	NB cell lines and patient samples show upregulated TKTL1 mRNA/protein versus controls. In a prospective pilot (*n* = 38 NB; *n* = 37 controls), 36/38 (94.7%) had EDIM‐TKTL1 >119; combined with Apo10, sensitivity 94.7% and specificity 100% for NB detection. No correlation with age, stage, MYCN status [142]	RMS cell lines (RD, RH30) and tumors show strong overexpression (up to 150‐fold mRNA in tumors). ROC analysis in 29 RMS versus 27 controls yielded a cut‐off >119, sensitivity 1.00 (95% CI 0.88–1.00), specificity 0.97 (0.83–1.00) [141]	Promoter hypomethylation may contribute to TKTL1 overexpression in RMS, supporting biological plausibility [141]
Apo10	Apo10 is an antibody epitope on DNaseX; its accumulation indicates dysregulated late‐stage apoptosis and aberrant proliferation [149], which characterize malignant cells Monocytes ingest these nuclear protein fragments, enabling intracellular detection [150]	In NB cell lines and tumors, Apo10 mRNA/protein is upregulated In the same pilot (*n* = 38 NB), 34/38 (89.5%) had EDIM‐Apo10 >129; combined TKTL1+Apo10 score (>248) detected 36/38 (94.7%) with specificity 100% versus controls [142]	Strong overexpression in tumors (up to 22.5‐fold protein). ROC in 29 RMS versus 27 controls gave a cut‐off >115, sensitivity 1.00, specificity 0.97; combined TKTL1/Apo10 cut‐off >238 also sensitivity 1.00, specificity 0.97 [141]	Apo10 complements TKTL1 by detecting an apoptosis–proliferation axis distinct from metabolic reprogramming, explaining their high combined diagnostic yield [141, 144]
GD2	GD2 is a surface ganglioside highly expressed in NB and some pediatric sarcomas, and it is extensively used as a diagnostic/therapeutic target [151] EDIM‐GD2 quantifies GD2 epitopes internalized by monocytes [152]	In a subset analysis (*n* = 19 NB; *n* = 22 controls), EDIM‐GD2 showed optimal ROC cut‐off of 12.95, sensitivity 78.95% (56.67%–91.49%), specificity 100% (85.13%–100.0%); 15/19 (79%) NB positive; 0% controls positive Some low‐level positivity appeared in non‐NB pediatric tumors (e.g., a minority of Wilms and sarcoma cases), consistent with known GD2 biology [142]	N/A	GD2 increases the diagnostic yield of TKTL1/Apo10 by adding tumor‐type information An expansion of EDIM panels to include additional tumor‐specific epitopes in other pediatric entities has been proposed [142]

Abbreviations: Apo10, DNaseX/Apo10 epitope; GD2, disialoganglioside; TKTL1, transketolase‐like 1; PPP, pentose phosphate pathway.

Exosomal (exo) noncoding RNAs (ncRNAs) are a promising biomarker class for pediatric MCED. Exosomes are small EVs that protect RNA from plasma RNases, so tumor‐derived ncRNAs (miRNAs, lncRNAs, circRNAs, other ncRNAs) can be recovered stably from blood. This makes them attractive analytes for repeatable, minimally invasive tests [153]. Pediatric tumors often have low TMBs compared with many adult cancers; hence, ctDNA‐based MCED approaches can be less sensitive. RNA signatures, including exo‐ncRNAs, capture transcriptional and cell‐state information that may reveal tumors without many point mutations [154]. Active clinical studies validating exosome‐based signatures include: (a) Saffari et al., who analyzed plasma exosomes from 30 pediatric B‐acute lymphoblastic leukemia (B‐ALL) patients and controls, measuring exo‐miR‐326 by qRT‐PCR as a potential diagnostic and prognostic marker [155]; (b) Tűzesi et al., who used primary pediatric high‐grade glioma cell lines to profile cellular and exosome‐derived miRNAs. By comparing glioma stem cells with neural fetal stem cells via microarrays and qRT‐PCR, they identified differentially expressed miRNAs, including miR‐1290 and miR‐1246, associated with stemness and invasiveness, suggesting exo‐miRNAs as candidate brain tumor biomarkers [156, 157]; and (c) Zhang et al., who provided early translational evidence supporting circulating exosomes as carriers of tumor‐derived signals in Ewing sarcoma [158]. The biological rationale and pilot data supporting the use of exo‐ncRNAs as tumor biomarkers for early cancer detection are encouraging, although major technical, clinical, and population‐level hurdles remain [159].

Additionally, genomic profiling techniques, such as virtual karyotyping, have been utilized to identify chromosomal abnormalities and detect submicroscopic changes, missed by traditional karyotyping, associated with pediatric cancers like neuroblastoma and Wilms' tumor [160, 161]. This method uses microarray‐based comparative genomic hybridization (aCGH) or single‐nucleotide polymorphism (SNP) arrays to detect copy number variations (CNVs) and loss of heterozygosity (LOH) across the genome, and unbalanced chromosomal rearrangements. Unlike traditional microscope‐based karyotyping, it does not require dividing cells, is faster, and provides much higher resolution. Virtual karyotyping, which can also be applied to archived tissue samples [162], facilitates early detection of unfavorable cytogenetics, supporting personalized treatment planning and risk stratification.

Table 7 provides details on ongoing clinical trials for each tumor type, with indication of cfDNA sources (blood/urine/CSF/bone marrow), age range of the recruited subjects, current trial phase (feasibility/cohort/randomized), and ClinicalTrials.gov identifiers (NCT numbers) for each tumor type.

**TABLE 7:** Active pediatric clinical trials of early cancer detection assays.

Tumor type	Age range	cfDNA/Liquid biopsy source	Current trial phase/Evidence	NCT number/Key references
Leukemia (ALL, AML)	ALL: ∼2–5 years; AML: all ages	Blood plasma/bone marrow cfDNA	Feasibility and cohort studies (MRD, relapse monitoring)	NCT06525116
CNS tumors (medulloblastoma, HGG)	All pediatric ages (e.g., 3–8 yrs for medulloblastoma)	CSF cfDNA (high yield); blood (lower sensitivity)	Feasibility and small prospective cohorts; diagnostic and monitoring trials	NCT05934630
Lymphomas (Hodgkin's, NHL)	School‐age to adolescence	Blood plasma cfDNA	Feasibility only; adult MCED includes lymphoma but lacks pediatric validation	[163]
Neuroblastoma	Infancy and early childhood (<2 years)	Blood plasma cfDNA; urine cfDNA	Cohort feasibility studies (risk stratification, relapse monitoring)	[164]
Wilms’ tumor (nephroblastoma)	Median ∼3–4 years	Blood plasma cfDNA; urine cfDNA	Feasibility and small cohort reports	NCT00002611
Rhabdomyosarcoma	Bimodal: ∼2–6 years and adolescents	Blood plasma cfDNA	Feasibility and early prognostic cohorts	NCT04625907
Ewing sarcoma/Osteosarcoma	Adolescents	Blood plasma cfDNA	Feasibility and cohort studies; relapse prediction	NCT06068075
Retinoblastoma	Infancy (<2 years)	Aqueous humor cfDNA (intraocular fluid); blood rarely	Case series/institutional feasibility only	[165]

*Notes*: All listed trials are feasibility or cohort‐driven; no randomized population MCED screening trials are available in pediatric oncology for these liquid biopsy approaches.

Abbreviations: ALL, acute lymphoblastic leukemia; AML, acute myeloid leukemia; CNS, central nervous system; CSF, cerebrospinal fluid; HGG, high‐grade glioma; MRD, minimal residual disease; NHL, non‐Hodgkin lymphoma.

Several groups and projects are leading the way in pediatric MCED‐related research. Notably, St. Jude Cloud, a cloud‐based data‐sharing ecosystem (https://www.stjude.cloud), and the Pediatric Cancer Genome Project (PCGP), a US‐based collaboration primarily between St. Jude Children's Research Hospital in Memphis, Tennessee, and Washington University School of Medicine in St. Louis, Missouri, have built a comprehensive genomic database. This resource currently hosts more than 1.25 petabytes of harmonized genomic data from over 10,000 pediatric cancer patients and survivors, including 12,104 whole‐genome, 7697 whole‐exome, and 2202 transcriptome sequences, providing an essential foundation to power future MCED algorithms [166]. The Children's Oncology Group (COG), an international organization with member institutions primarily in the United States, Canada, Australia, and New Zealand, as well as sites in Europe and the Middle East [167], is exploring liquid biopsy applications using ctDNA for pediatric cancer monitoring and early detection, including in patients with newly diagnosed Ewing sarcoma, osteosarcoma [168], and Wilms tumor [169]. Similarly, the INdividualized therapy FOr Relapsed Malignancies in Childhood (INFORM) Registry in Germany is a registry‐based molecular profiling program for children and young adults with relapsed or refractory high‐risk cancers lacking effective standard therapies. INFORM applies comprehensive NGS, including whole‐exome, low‐coverage whole‐genome, RNA‐seq, and methylation profiling, to generate tumor‐specific molecular fingerprints reviewed by an expert multidisciplinary panel for clinical relevance [170].

Future development of MCED tests in children may enable earlier detection of aggressive tumors, improved surveillance for recurrence in survivors, and enhanced risk stratification for hereditary cancer syndromes such as Li‐Fraumeni [171], building on demonstrated benefits in adults [172, 173]. However, robust clinical trials, rigorous biomarker validation, and clear ethical frameworks are required before routine pediatric implementation.

## 8 Conclusions, Challenges, and Prospects

MCED tests represent a breakthrough in cancer diagnosis, bridging the gap between tumor onset and clinical manifestation through advanced technologies powered by AI algorithms. ML, particularly deep learning using artificial neural networks, identifies patterns in large datasets by integrating multiple biological layers (multi‐omics), including genomics, transcriptomics, proteomics, methylation data, and metabolic signatures, that correlate with different cancers. This approach enables early, noninvasive detection of a range of malignancies through a safe blood test, before symptoms appear [174].

Building on a review of key milestones in MCED technology, this article examines how AI systems enhance diagnostic and predictive capabilities, explores molecular mechanisms that limit early cancer detection, and highlights innovative detection platforms designed to overcome these challenges. Ongoing clinical trials and validation efforts aimed at facilitating the clinical adoption of MCED tests are summarized. The review also provides an overview of pediatric applications, emphasizing ongoing challenges and prospective solutions for wider implementation.

Overall, although promising for population‐wide cancer screening, several outstanding questions across scientific, clinical, regulatory, economic, health equity, and ethical dimensions have yet to be answered before MCED tests can be broadly implemented and useful in real‐world settings. Major issues are addressed in the following sections.

### 8.1 Scientific and technical challenges, such as sensitivity and specificity, cancer type coverage, stage‐specific performance, and biological variability

MCED tests must balance early cancer detection (sensitivity) with minimizing false positives and negatives (specificity). Detection performance varies across cancer types, with pancreatic, ovarian, liver, kidney, and brain cancers being particularly challenging. Transparent reporting of sensitivity and specificity by histopathological and molecular subtype is essential. To address these disparities, regulatory and research agencies should support assay refinement, establish minimum performance thresholds, and consider clinical utility relative to available treatment options. MCED tests may perform better at later disease stages, so clear communication about limitations is critical to manage patient expectations and reduce anxiety. Individual factors, such as age, genetics, lifestyle, and family history, also affect accuracy. Integrating genetic risk profiles, adjusting detection thresholds, and validating algorithms in diverse populations are key to optimizing predictive value, reducing bias, and ensuring equitable clinical application [175].

### 8.2 Healthcare system barriers, follow‐up procedures, and healthcare infrastructure

MCED tests need to complement, not replace, existing cancer screening protocols [176]. There is no consensus on how MCED tests should be used alongside existing cancer screenings to impact survival and long‐term outcomes [115]. AI and ML should be used to support the execution of this supposed synergistically integrated strategy, ensuring that clinics are equipped to handle increased testing and follow‐up demands. Healthcare providers must be educated on how to interpret and act on MCED results.

### 8.3 Ethical, social, and behavioral concerns, which include risk of overdiagnosis and overtreatment, the need for public acceptance and compliance

Detecting slow‐growing or nonlethal cancers can lead to unnecessary interventions, while patients may hesitate due to fear of false positives or uncertainty about follow‐up care. Healthcare professionals need training to provide culturally sensitive counseling and decision‐making tools that clearly explain benefits, risks, and required follow‐up. Policies should balance the benefits of early detection with the risks of false positives, invasive procedures, and psychological distress. Effective screening requires systematic tracking of outcomes through registries, integration of patient‐reported measures to capture psychological and quality‐of‐life impacts, and standardized reporting of diagnostic complications. Regular evaluation of these data can guide adjustments to eligibility criteria, screening intervals, or follow‐up protocols when potential harms outweigh benefits. These measures protect patients and reinforce public trust by promoting transparency and evidence‐based practice [177].

### 8.4 Regulatory and policy considerations, such as approval and standardization, data privacy, and security

Regulatory agencies need clear guidelines on how to evaluate and approve MCED tests. Handling of large‐scale genetic and biomarker data raises concerns about patient privacy and consent. To address these issues, patient advocacy groups should be involved early in shaping policies and consent models. It is advisable to establish independent oversight boards, including patient representatives, to review data use and sharing requests, and to develop international standards aligned with the General Data Protection Regulation (GDPR) for the EU, or with the Health Insurance Portability and Accountability Act (HIPAA) for the USA (or other regulatory agencies), to ensure personal data protection rights and to handle global research collaborations safely.

### 8.5 Economic and industry factors, which include the cost of testing, industry competition, and quality control

The development of MCED tests by multiple companies may lead to variability in quality and effectiveness, requiring oversight by health authorities. High costs (retail prices currently around $900–$1000 and largely unreimbursed) limit adoption, particularly in low‐resource settings, concentrating benefits among higher‐income groups. Most insurers do not cover screening, and follow‐up diagnostic costs are often uncertain. Policy measures to reduce inequities include funding regional pilot programs that provide free MCED testing to underserved populations within structured monitoring frameworks. Key actions involve negotiating test prices and subsidies through national health systems, insurers, and social health funds, supporting assistance programs, and avoiding direct‐payment models that disproportionately favor wealthier patients [175].

A robust collaborative network of researchers, policymakers, healthcare providers, and the public is essential to ensure MCED tests are effective, accessible, and ethically implemented [115]. Ongoing research aims to refine these technologies, improve detection rates, and support integration into healthcare systems.

Combining MCED tests with advanced molecular imaging represents a strategic innovation with the potential to redefine cancer screening paradigms, as early feasibility studies indicate this approach can enhance cancer detection beyond current standard screening methods [178, 179]. MCED tests can act as a broad triage/signal that a cancer is present and eventually predict TOO. When positive, the next step is a focused diagnostic cascade, by using targeted imaging rather than indiscriminate biopsies. Whole‐body molecular imaging, such as FDG‐PET/CT, PET/MRI, or newer receptor/targeted PET tracers, is the most practical and evidence‐backed next step to locate otherwise occult tumors.

Near‐term growth will be driven by advances in liquid biopsy (ctDNA, EVs, CTCs), novel molecular imaging, including PET/SPECT tracers beyond 18F‐FDG, such as prostate‐specific membrane antigen (PSMA), fluoroestradiol (FES), amino‐acid and hypoxia tracers, that detect lesions earlier or at smaller sizes [180], and computational biomarkers (radiomics/AI) that enhance detection and localization [181], ultimately improving sensitivity and clinical utility. Widespread clinical use hinges on improved sensitivity/specificity, localization capability, cost/reimbursement, and clear regulatory pathways [182].

In conclusion, key technological trends shaping future development involve: (a) Higher‐sensitivity liquid biopsy methods, which exploit deep whole‐genome sequencing, methylation signatures, and fragmentomics, improving early‐stage detection and MRD monitoring [183]. (b) Targeted PET tracers and theranostics, using proliferation or target‐specific radiotracers (PSMA, FES, novel receptor ligands), enabling earlier biological detection and therapy pairing [180]. (c) Total‐body and large axial‐field PET scanners, which increase sensitivity and reduce radiation dose, are important for detecting very small/low‐uptake lesions [184]. (d) AI, radiomics, and multi‐omics integration to extract weak signals by combining imaging, liquid biopsy and clinical data, enabling actionable clinical decisions [185, 186].

**Evidence highlights and limitations**: Studies and pilot programs, including PATHFINDER, show that MCED tests can detect a cancer signal, and whole‐body imaging often identifies the lesion, though sensitivity varies by cancer type and stage. Ongoing registrational trials and multimodal cfDNA analyses, incorporating methylation, fragmentation, and tissue‐specific signals, improve TOO prediction to guide imaging, though localization accuracy remains platform‐ and cancer‐dependent [179, 187].

**A critical open question remains**: *To what extent do MCED‐triggered imaging and diagnostic strategies reduce cancer mortality or enhance quality‐adjusted life years in a cost‐effective manner?* There is a recognized need for randomized or well‐designed prospective studies to demonstrate clinical utility and cost‐effectiveness [12]. These studies can be strengthened by specific research and implementation programs.

**Research and implementation priorities include: (a) Standardized diagnostic algorithm trials**. Randomized or prospective cohort studies comparing MCED and imaging cascade versus standard‐of‐care diagnostic pathways, with endpoints: time‐to‐diagnosis, stage at diagnosis, diagnostic yield, treatment changes, mortality, harms, and cost‐effectiveness [176]. **(b) Tracer‐selection algorithms**. Studies that use MCED‐derived TOO signals to guide the selection of the most appropriate imaging tracer (e.g., determining when FDG, PSMA, DOTATATE or FAPI is most suitable. Retrospective linking of cfDNA predictions with imaging and biopsy outcomes is used to train decision‐making rules [179]. **(c) False‐positive management protocols**, which establish thresholds, such as TOO confidence and cfDNA signal strength, that determine whether immediate imaging or watchful waiting is warranted, minimizing overdiagnosis. **(d) Health‐economic analyses**, which model costs at scale for population‐wide screening versus targeted high‐risk use, accounting for downstream costs of imaging and biopsies [188]. **(e) Registry and real‐world data collection**, consisting of centralized registries to capture MCED positives, imaging performed, final diagnoses, and outcomes to quickly refine best practices.

Over the next 5–10 years, advances in liquid biopsy, novel tracers, total‐body PET, and AI are expected to drive substantial technical progress, with near‐term clinical benefits in MRD detection and targeted localization. While population‐wide screening will require rigorous validation to limit harms from false positives, early pilot programs and emerging theranostic strategies show encouraging potential [189]. MCED assays and advanced molecular imaging represent a complementary paradigm, in which MCED identifies individuals warranting further evaluation and molecular imaging enables anatomical and biological localization. Early feasibility studies and real‐world implementations suggest improved diagnostic efficiency, but robust evidence for long‐term clinical benefit and cost‐effectiveness remains limited. Future progress will depend on well‐designed clinical trials, optimized tracer selection, and cautious implementation supported by multidisciplinary oversight.

## Author Contributions

Emma Di Carlo: Data curation, writing – original draft, writing – review and editing.

## Funding

This work was supported by Associazione Italiana per la Ricerca sul Cancro (AIRC), IG 2024 ‐ ID. 30316 project, P.I. Emma Di Carlo; European Union—NextGenerationEU, Ministero della Salute, PNRR Program, Project “From inflammatory bowel disease to colon cancer: involvement of innate lymphocytes in early pathogenic mechanisms”, ID. PNRR‐MAD‐2022‐12375909, Head of Local Operative Unit—Emma Di Carlo.

## Ethics Statement

The authors have nothing to report.

## Conflicts of Interest

The author declares no conflicts of interest.

## Supporting information




**Table S1**: Mechanisms of cancer cell resistance to apoptosis across common tumor types.
**Table S2**: Preclinical studies supporting MCED method development.
**Table S3**: Two‐tier evidence–based MCED development programs.
**Table S4**: Tailored early‐detection strategies for hard‐to‐detect cancers.

## Data Availability

The author has nothing to report.
