# Supplementary material for: Early Cancer Detection: What's Going on and What's Next
Source: MedComm (2020). 2026 Mar 11;7(3):e70653. doi: 10.1002/mco2.70653 (PMC13097591; doi:10.1002/mco2.70653)
Supplement: Supplementary file 1 — Table S1: Mechanisms of cancer cell resistance to apoptosis across common tumor types. Table S2: Preclinical studies supporting MCED method development. Table S3: Two‐tier evidence–based MCED development programs. Table S4: Tailored early‐detection strategies for hard‐to‐detect cancers. [file MCO2-7-e70653-s001.docx]

**Early Cancer Detection: What's Going on and What's Next**

Emma Di Carlo^1,2^

^1.^ Department of Medicine and Sciences of Aging, "G. d'Annunzio" University” of Chieti-Pescara, Via dei Vestini, Chieti, 66100, Italy.

^2.^ Anatomic Pathology and Immuno-Oncology Unit, Center for Advanced Studies and

Technology (CAST), "G. d'Annunzio" University of Chieti-Pescara, Via L. Polacchi 11,

Chieti, 66100, Italy.

**Supplementary Table S1.** Mechanisms of Cancer Cell Resistance to Apoptosis Across Common Tumor Types

| **Mechanism** | **Molecular players / Examples** | **Reference tumor types** | **Description** | **Key References** |
| --- | --- | --- | --- | --- |
| **Overexpression of anti-apoptotic Bcl-2 family proteins** | Bcl-2, Bcl-XL, Mcl-1 | Follicular lymphoma, CLL, breast cancer. | Prevents mitochondrial outer membrane permeabilization (MOMP) and cytochrome c release. | *Nat Rev Cancer.* 2002 Sep;2(9):647-56.  doi: 10.1038/nrc883. |
| **Loss or mutation of pro-apoptotic proteins** | Bax, Bak, Bid, Puma, Noxa | Colorectal cancer, glioblastoma, breast cancer. | Blocks intrinsic mitochondrial apoptosis signaling. | *Cell.* 2009 May 1;137(3):413-31. doi: 10.1016/j.cell.2009.04.037. |
| **Loss or inactivation of p53** | TP53 mutations, MDM2 amplification | Lung cancer, ovarian cancer, sarcomas. | Disables DNA damage–induced apoptosis and cell cycle arrest. | *Cell.* 1997 Feb 7;88(3):323-31.  doi: 10.1016/s0092-8674(00)81871-1. |
| **PTEN loss/inactivation** | PTEN mutation, deletion, promoter methylation | Glioblastoma, prostate cancer, endometrial cancer. | Removes inhibition of PI3K/AKT pathway, enhancing survival signaling and resistance to apoptosis. | *Cell.* 2008 May 2;133(3):403-14. doi: 10.1016/j.cell.2008.04.013.  *Nat Rev Mol Cell Biol.* 2018 Sep;19(9):547-562.  doi: 10.1038/s41580-018-0015-0. |
| **Overactivation of survival signaling pathways** | PI3K/Akt, NF-κB, MAPK | Pancreatic cancer, glioblastoma, prostate cancer. | Promotes proliferation and suppresses apoptosis. | *Cancer Treat Rev.* 2004 Apr;30(2):193-204.  doi: 10.1016/j.ctrv.2003.07.007. |
| **Reduced expression or mutation of death receptors** | Fas (CD95), TRAIL-R1/2 | Melanoma, hepatocellular carcinoma. | Blocks extrinsic apoptotic signaling. | *Cell Death Differ.* 2003 Jan;10(1):26-35.  doi: 10.1038/sj.cdd.4401186. |
| **Upregulation of decoy receptors** | DcR1, DcR2, osteoprotegerin | Colon cancer, lung cancer. | Competes with functional death receptors for ligands (FasL, TRAIL). | *Nat Rev Cancer.* 2002 Jun;2(6):420-30.  doi: 10.1038/nrc821. PMID: 12189384.  *Cell Death Differ.* 2003 Jan;10(1):66-75.  doi: 10.1038/sj.cdd.4401187. |
| **Overexpression of IAPs (Inhibitor of Apoptosis Proteins)** | XIAP, Survivin, c-IAP1/2 | Leukemia, colorectal cancer, gastric cancer. | Inhibits caspase activation and blocks apoptosis execution. | *Nat Rev Drug Discov.* 2012;11:331.  doi: 10.1038/nrd3698. |
| **FLIP overexpression** | c-FLIP (FLICE-inhibitory protein) | Melanoma, cervical cancer. | Prevents caspase-8 activation at the DISC (death-inducing signaling complex). | *Nature.* 1997 Jul 10;388(6638):190-5.  doi: 10.1038/40657.  *FEBS J.* 2018 Nov;285(22):4104-4123.  doi: 10.1111/febs.14523. |
| **Altered caspase expression or function** | Caspase-8, Caspase-9 downregulation/mutation | Neuroblastoma, head and neck squamous cell carcinoma. | Inhibits initiation/execution of apoptosis. | *Cell Death Differ.* 2011 Sep;18(9):1441-9.  doi: 10.1038/cdd.2011.30. |
| **Autophagy-mediated resistance** | Beclin-1 modulation, LC3 upregulation | Glioblastoma, pancreatic cancer. | Provides alternative survival mechanism under stress, bypassing apoptosis. | *Nat Rev Cancer.* 2012 Apr 26;12(6):401-10.  doi: 10.1038/nrc3262. |
| **Dysregulation of lncRNAs** | NEAT1, MALAT1, HOTAIR, GAS5 | Breast cancer, hepatocellular carcinoma, glioma, colorectal cancer, lung cancer, gastric cancer. | LncRNAs regulate apoptosis by sponging tumor-suppressive miRNAs, modulating p53/Bcl-2 pathways, or altering caspase activity. | *Cancer Res.* 2017 Aug 1;77(15):3965-3981.  doi: 10.1158/0008-5472.CAN-16-2634.  *Cancer Cell Int.* 2025 Apr 1;25(1):123.  doi: 10.1186/s12935-025-03679-0. |

**Supplementary Table S2.** Preclinical Studies Supporting MCED Method Development

| **Test / Group** | **Year** | **Modality**  **(biomarker)** | **Preclinical model / Data used** | **Key preclinical**  **results** | **Key References** |
| --- | --- | --- | --- | --- | --- |
| **CancerSEEK (Johns Hopkins)** | 2018 | Multianalyte: somatic mutations (ctDNA) + protein markers. | Archived human plasma from cases & controls; analytic assay development in lab. | Demonstrated proof-of-concept that combining mutation and protein signals can detect multiple tumor types with high specificity in case-control sets; informed assay design for DETECT-A trial. | *J Natl Cancer Inst.* 2023 Mar 9;115(3):250-257.  doi: 10.1093/jnci/djac218. |
| **Targeted cfDNA methylation (GRAIL / CCGA initial discovery)** | 2019–2020 (CCGA substudy posters/papers) | Targeted genome-wide cfDNA methylation. | Large archived/clinical plasma sets (discovery & training on thousands of samples), analytic optimization (in-lab). | Genome-wide methylation patterns gave strong cancer vs non-cancer signal and tissue-of-origin information in discovery and internal validation sets — supported selection of methylation panel for clinical development. | *Ann Oncol.* 2020 Jun;31(6):745-759.  doi: 10.1016/j.annonc.2020.02.011. |
| **Targeted methylation MCED (analytical validation / early clinical validation)** | 2021 | Targeted methylation (cfDNA). | Assay analytic validation on clinical samples and case-control cohorts. | Reported high specificity with the ability to detect a wide range of cancer types and predict tissue-of-origin in validation cohorts (supporting transition from preclinical to clinical validation). | *Ann Oncol.* 2021 Sep;32(9):1167-1177.  doi: 10.1016/j.annonc.2021.05.806. |
| **PapSEEK (Johns Hopkins - tumor DNA in cervical/vaginal samples) / discovery phase** | 2018 (preclinical/analytic work reported with early cohorts) | Tumor DNA (mutations) from cervical/vaginal fluid + targeted sequencing. | Archived patient samples, assay optimization in lab. | Showed the concept that tumor DNA could be detected in routine gynecologic samples with high specificity — informed assay design for screening of gynecologic cancers. | *Sci Transl Med.* 2018 Mar 21;10(433):eaap8793.  doi: 10.1126/scitranslmed.aap8793. |
| **Reviews / framework for preclinical development of MCED tests** | 2022 | Methodology/framework. | Synthesis of discovery → assay development steps using published preclinical examples. | Lays out a 5-step preclinical→translational path (discovery → assay development → analytic validation → clinical validation → implementation), highlighting common practices and pitfalls in preclinical MCED work. | *Cancer.* 2022 Feb 15;128 Suppl 4(Suppl 4):861-874.  doi: 10.1002/cncr.33912. |
| **Other biomarker discovery (miRNA/other multi-analyte platforms; earlier-stage)** | 2014–2020 (representative) | microRNA, protein panels, other cfDNA features. | Cell lines, archived plasmas, small case-control cohorts. | Multiple groups reported promising discrimination in case-control sets, but limited prospective/analytic validation, highlighting heterogeneity of preclinical evidence. | *Methods Mol Biol.* 2012;822:19-31.  doi: 10.1007/978-1-61779-427-8_2.  *Nat Rev Genet.* 2012 Apr 18;13(5):358-69.  doi: 10.1038/nrg3198. |

**Supplementary Table S3.** Two-Tier Evidence–Based MCED Development Programs

| **Program** | **Tier 1**  **(Performance / Registrational)** | **Tier 2**  **(Clinical Utility / Pragmatic RCTs)** |
| --- | --- | --- |
| **GRAIL (Galleri®)** | *PATHFINDER-2:* large interventional study (N≈35,000 in the U.S.) evaluating diagnostic yield, accuracy, and integration of Galleri into clinical pathways.  *Case–control and discovery studies* (CCGA, SUMMIT, STRIVE) that established analytic validity and biomarker signal performance. | *NHS-Galleri Trial (UK)*: ~140,000 participants, double-blind RCT with late-stage cancer incidence as the primary endpoint. Results expected in 2026. |
| **Exact Sciences** | *Falcon Registry*: prospective, multi-site real-world evidence (RWE) registry enrolling 25,000 participants, with comparator cohort of ~50,000. Focuses on test accuracy, patient/provider experience, and workflow integration.  Ongoing discovery and validation studies of their investigational MCED platform. | Planning pragmatic trials to assess stage shift and outcomes, though definitive RCTs have not yet launched. |
| **Guardant Health** | Validation studies of *Shield*™, initially focused on colorectal cancer but extended into multicancer detection research. | *NCI CSRN Vanguard Study*: Shield selected as one of two MCED platforms tested in a pilot RCT (with Avantect, ClearNote Health), randomizing ~24,000 participants to assess feasibility, adherence, and readiness for a larger mortality-endpoint trial. |
| **ClearNote Health** | Development studies of *Avantect*®, an epigenomic cfDNA-based MCED test. | *NCI CSRN Vanguard Study*: Avantect is the second platform being tested alongside Shield, under the same randomized feasibility design. |
|  |  |  |

**Supplementary Table S4.** Tailored Early-Detection Strategies for Hard-to-Detect Cancers

| **Cancer Type** | **Key Challenges** | **Tailored Solutions** |
| --- | --- | --- |
| **Pancreatic** | Low ctDNA shedding  in early disease.  Rapid progression,  often diagnosed late. | Use multimodal assays (methylation, fragmentomics and protein markers).  Prioritize pancreas-protocol CT/MRI for positives.  Referral to specialized pancreatic centers for surgical evaluation. |
| **Ovarian** | Nonspecific symptoms, often detected at advanced stage.  Lack of established population screening. | Combine MCED positives with transvaginal ultrasound and CA-125 reflex testing.  Ensure rapid gynecologic oncology referral.  Capture histologic subtypes  (especially high-grade serous) in trials. |
| **Liver** | High stage dependency  for outcomes.  Existing screening only in high-risk groups (cirrhosis, hepatitis B).  Risk of false positives in general population. | Deploy MCED as adjunct in high-risk groups and to detect hidden at-risk patients (e.g., Non-Alcoholic Fatty Liver Disease, NAFLD).  Positive results → targeted liver imaging (ultrasound/CT/MRI) and hepatology referral. Integrate with viral hepatitis and liver disease monitoring. |
| **Kidney** | Many cases found incidentally.  Some tumours indolent  (risk of overtreatment). | Use MCED for accurate tissue-of-origin assignment to identify renal origin.  Positive results → contrast CT/MRI of kidneys.  Balance nephron-sparing surgery vs active surveillance in diagnostic pathway. |
| **Brain** | Blood–brain barrier limits ctDNA detection.  Symptoms appear only after significant progression. | Investigate CSF-based biomarkers as complement to blood tests.  Positive MCED brain signal → rapid neuroimaging (MRI/CT).  Immediate neuro-oncology referral to guide next steps. |
